# A scoping review of global COVID-19 vaccine hesitancy among pregnant persons

**DOI:** 10.1038/s41541-024-00913-0

**Published:** 2024-07-20

**Authors:** Imaima Casubhoy, Alyssa Kretz, Heang-Lee Tan, Laura A. St Clair, Maclaine Parish, Hana Golding, Susan J. Bersoff-Matcha, Catherine Pilgrim-Grayson, Leah Berhane, Andrew Pekosz, Heba H. Mostafa, Andrea L. Cox, Irina Burd, Sabra L. Klein, Rosemary Morgan

**Affiliations:** 1 Department of International Health, Johns Hopkins University Bloomberg School of Public Health, Baltimore, MD USA; 2 W. Harry Feinstone Department of Molecular Microbiology and Immunology, Johns Hopkins University Bloomberg School of Public Health, Baltimore, MD USA; 3 Division of Viral Products, Center for Biologics Evaluation and Research, U.S. Food and Drug Administration, Silver Spring, MD USA; 4 Office of Women’s Health, U.S. Food and Drug Administration, Silver Spring, MD USA; 5 Office of Rare Diseases, Pediatrics, Urologic and Reproductive Medicine-Division of Urology, Obstetrics, and Gynecology, Office of New Drugs, Center for Drug Evaluation and Research, U.S. Food and Drug Administration, Silver Spring, MD USA; 6 Department of Medicine, Johns Hopkins University School of Medicine, Baltimore, MD USA; 7 Department of Pathology, Division of Medical Microbiology, Johns Hopkins University School of Medicine, Baltimore, MD USA; 8 Department of Obstetrics, Gynecology and Reproductive Sciences, University of Maryland School of Medicine, Baltimore, MD USA

**Keywords:** Public health, Viral infection

## Abstract

Uptake of the COVID-19 vaccine among pregnant persons is lower than the general population. This scoping review explored pregnant people’s attitudes towards the COVID-19 vaccine, reasons for vaccine hesitancy, and whether attitudes about COVID-19 vaccines differ by country of origin. A scoping review was conducted across PubMed, Embase, CINHAL, and Scopus. Inclusion criteria were articles published in English from 2019–2022 focused on attitudes towards COVID-19 vaccination among pregnant persons. Data analysis was done via the 5Cs framework for vaccine hesitancy: Constraints, Complacency, Calculation, Confidence, and Collective Responsibility. 44 articles were extracted. A lack of confidence in vaccine safety was the most prevalent theme of hesitancy among pregnant persons. This was largely driven by a lack of access to information about the vaccine as well as mistrust of the vaccine and medical professionals. Meanwhile, vaccine acceptance was mostly driven by a desire to protect themselves and their loved ones. Overall, COVID-19 vaccine hesitancy among pregnant persons continues to be high. Vaccine hesitancy is primarily driven by fear of the unknown side effects of the vaccine on pregnant persons and their fetuses along with a lack of information and medical mistrust. Some differences can be seen between high income and low- and middle-income countries regarding vaccine hesitancy, showing that a single solution cannot be applied to all who are vaccine hesitant. General strategies, however, can be utilized to reduce vaccine hesitancy, including advocating for inclusion of pregnant persons in clinical trials and incorporating consistent COVID-19 vaccine counseling during prenatal appointments.

## Introduction

The COVID-19 pandemic has caused a significant change in the landscape of healthcare on a global level. As of April 2023, there have been a total of 764,474,387 cases of COVID-19 globally, with a total of 6,915,286 deaths^1^. When stratified by WHO defined regions, Europe had the highest number of confirmed cases (275,789,453), whereas Africa had the fewest cases (9,522,906)^1^. Based on COVID-19 deaths, the Americas had the highest number of COVID-19 deaths (2,945,996) and Africa had the fewest (175,343)^1^. In response to the rapid spread of the COVID-19 virus, the development and distribution of the COVID-19 vaccine was also expedited on a global level. Thus, it has become even more important for researchers and practitoners to better understand vaccine hesitancy in the lens of a pandemic.

The World Health Organization (WHO) defines vaccine hesitancy as a “delay in acceptance or refusal of vaccination, despite availability of vaccination services”^2^. As of 2019, vaccine hesitancy was identified as one of the top 10 global health threats, as identified by WHO^3^. The concept of vaccine hesitancy has been a highly studied topic globally, with multiple studies that have examined vaccine hesitancy in both childhood and adulthood for a variety of diseases ranging from influenza to the measles, mumps and rubella (MMR)^4^. Globally, vaccination uptake has been relatively high, with 13,325,228,015 vaccine doses administered. Of these doses, 72.3% (5,546,760,991) of recipients have received at least one vaccine dose and 67% (5,104,768,538) of recipients received a full primary series of doses, which differs depending on the vaccine platform^1,5^. When looking at global vaccination trends, it is important to address the inequity that exists in vaccine availability. Production and availability of vaccines is affected by the economic status of a country, resulting in decreased availability in many LMICs^6^. However, COVID-19 prevalence, mortality, and vaccination rates have all been difficult to determine for at risk populations, such as pregnant persons.

Per the CDC, it is estimated that 71.4% of pregnant persons have completed the primary COVID-19 vaccine series and 22.9% have received updated booster doses in the United States. The global rate of COVID-19 vaccination among pregnant persons, however, is estimated to only be 53.4%^7^. It is crucial to address vaccine hesitancy among pregnant people, as they are at a higher risk for severe consequences from COVID-19 compared to nonpregnant counterparts. Pregnant persons who get COVID-19 are at greater risk for complications, including hospitalization, admission into intensive care units (ICU), intubation, and death^8^. Moreover, pregnant patients who have a symptomatic COVID-19 disease course have nearly three times the odds of developing adverse maternal outcomes (e.g. ICU admission, invasive ventilation, preterm delivery, death) than pregnant patients who do not have COVID-19^8^. There also is a discrepancy regarding the approach to COVID-19 vaccine policies among different countries. There are 121 countries that explicitly recommend COVID-19 vaccination of pregnant persons, with 64 additional countries that allow pregnant persons to receive the COVID-19 vaccination, but do not have official recommendations^9^. Finally, there are 9 countries that in which certain groups of pregnant persons, such as healthcare workers and pregnant persons with chronic conditions, can receive the vaccine, but no explicit recommendation is issued. One country (Cote d’Ivoire) that does not recommend COVID-19 vaccination, with exceptions, and nine countries that do not recommend COVID-19 vaccines at all for pregnant persons.

The exclusion of pregnant persons from COVID-19 vaccine trials likely have affected vaccine uptake among this group^10^. In general, pregnant persons have been excluded from 69% of all clinical trials in the U.S., with the COVID-19 vaccine clinical trials being no exception^11^. Due to this, initial recommendations were that pregnant persons should not receive the vaccine until more data were gathered. These initial recommendations were also influenced by an initial lack of evidence that pregnant persons were at high risk for severe illness secondary to a COVID-19 infection. Early in January 2021, the prevailing opinion of professional organizations such as the WHO, the American College of Obstetrics and Gynecology (ACOG), and the Society for Maternal-Fetal Medicine was that vaccination during pregnancy was an individual’s choice and was not uniformly recommended to all pregnant persons^12^. These recommendations changed as information about COVID-19 vaccine efficacy and safety as well as evidence highlighting that pregnant persons as a high-risk population were published through the course of the pandemic, with COVID-19 vaccines currently being highly recommended for pregnant persons in the US.

Vaccination rates among pregnant persons are currently estimated to be lower than those of the general population^7^, implying that pregnant persons are among the most hesitant to receive the vaccine^13^. This scoping review aims to explore pregnant people’s attitudes towards the COVID-19 vaccine, reasons for vaccine hesitancy, and whether attitudes about COVID-19 vaccines differ by country of origin. In addition, this review will provide guidance on how to address vaccine hesitancy in this special-risk group, which could potentially increase uptake.

## Results

**Fig. 1:**
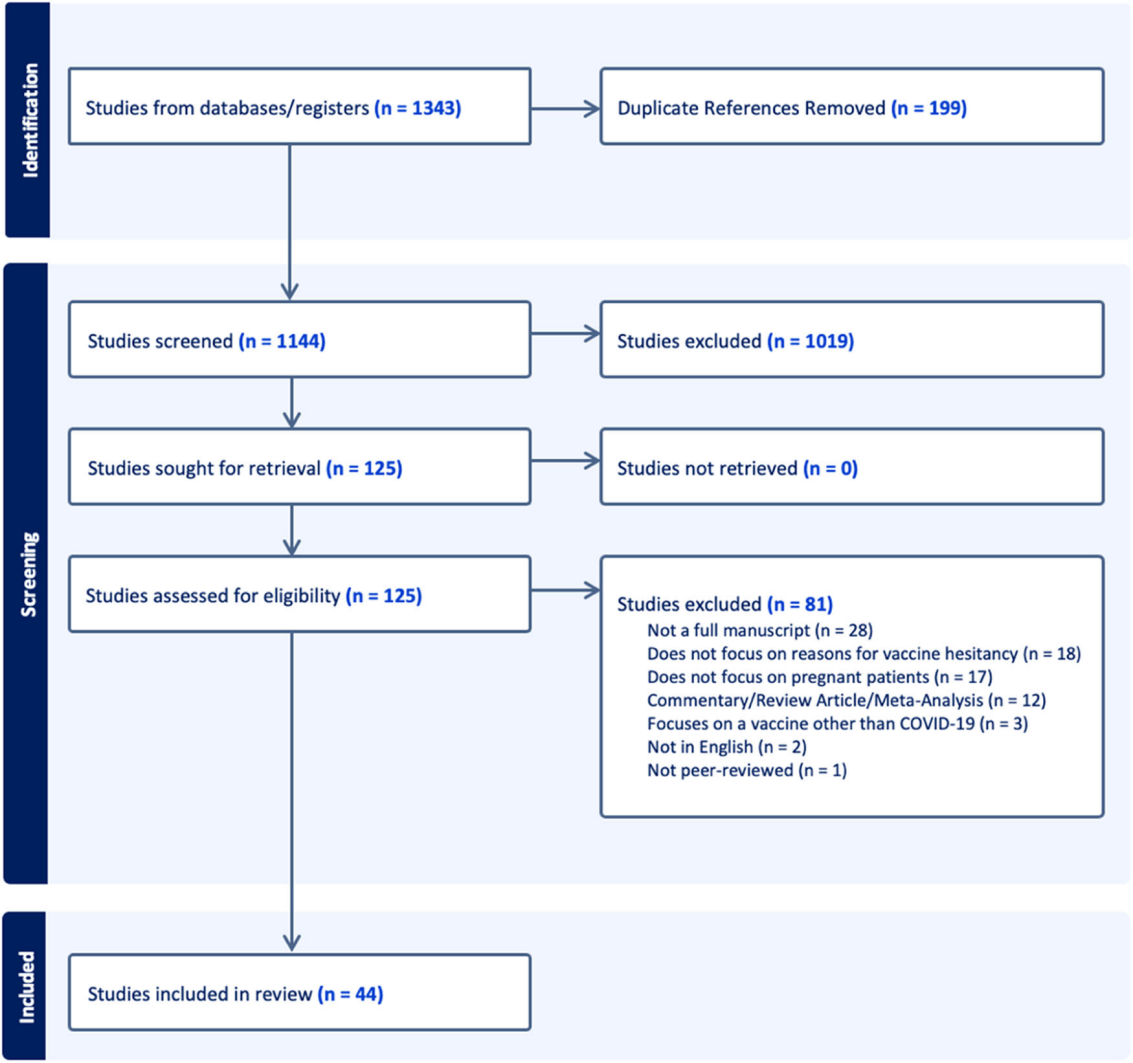
PRISMA chart. PRISMA chart results.

The final search strategy, modified for each database, resulted in a total of 1343 citations that were uploaded to Covidence. After 199 duplicate citations were removed, a reminder of 1144 citations were screened by title and abstract, resulting in 125 studies being eligible for full text review. A final count of 44 studies were extracted for analysis. The detailed search process is illustrated via the PRISMA chart in Fig. 1.

### Flowchart for article retrieval process

Among the 44 included studies, 38 were quantitative, one was qualitative, and five were mixed methods. A total of 40,934 participants were included from 39 different countries: thirteen countries in Europe, six countries in the Western Pacific Region, eight countries in the Americas, five countries in Africa, four counties in the Eastern Mediterranean region, and three countries in South-East Asia (Table 1).

**Table 1:** Summary of extracted articles

Study	Country	Study type	No. participants	Study timeline	Number (%) vaccine hesitant
High Income Countries (HIC)					
Davies et al.^55^	England	Quantitative	202	October–November 2021	39 (33%)
Rikard-Bell et al.^17^	Australia	Quantitative	287	September 15–October 22, 2021	25%
Geoghegan et al.^24^	Ireland (HIC)	Mixed-Methods	300	December 4, 2020–January 14, 2021	108 (36%)
Sutton et al.^30^	USA (HIC)	Quantitative	1012	January 7–29, 2021	108 (50%)
Battarbee et al.^33^	USA (HIC)	Quantitative	914	August 9–December 10, 2020	429 (46.9%)
Perrotta et al.^48^	USA (HIC)	Quantitative	299	March 1–July 23, 2021	82 (27.4%)
Schaal et al.^46^	Germany (HIC)	Quantitative	2339	March 30–April 19, 2021	878 (86.3%)
Reifferscheid et al.^35^	Canada (HIC)	Quantitative	193	May 28–June 7, 2021	82 (42.6%)
Husain et al.^29^	UK (HIC)	Quantitative	441	September 1, 2021–February 28, 2022	Not reported
Citu et al.^50^	Romania (HIC)	Quantitative	345	October 1–December 1, 2021	96 (52.2%)
Razzaghi et al.^40^	USA (HIC)	Quantitative	1516	March 31–April 16, 2021	54.30%
Levy et al.^14^	USA (HIC)	Quantitative	662	December 14, 2020–January 14, 2021	277
Simmons et al.^51^	USA (HIC)	Quantitative	387	December 24, 2020–January 27, 2021	220 (57%)
Mattocks et al.^36^	USA (HIC)	Quantitative	72	January–May 2021	50 (69%)
Ghamri et al.^42^	Saudi Arabia (HIC)	quantitative	5307	July–September 2021	32%
Hosokawa et al.^53^	Japan (HIC)	Quantitative	1621	July 24–August 30, 2021	825 (50.9%)
Citu et al.^43^	Romania (HIC)	Quantitative	345	January 1–May 1, 2022	Not reported
Nowacka et al.^28^	Poland (HIC)	Quantitative	1033	December 2021–February 2022	324 (31.4%)
Egloff et al.^22^	France (HIC)	Quantitative	664	February 18–April 5, 2021	468 (70.5%)
Ward et al.^34^	Australia (HIC)	Quantitative	218	September–October 2021	36 (29.5%)
Tatarević et al.^37^	Croatia (HIC)	Quantitative	430	May–October 2021	361 (84%)
Colciago et al.^25^	Italy (HIC)	Quantitative	538	February 1–March 3, 2022	17.30%
DesJardin et al.^44^	USA (HIC)	Quantitative	157	September–October 2021	97 (61.7%)
Sutanto et al.^23^	USA (HIC)	Quantitative	109	August 6–September 10, 2021	35
Redmond et al.^45^	USA (HIC)	Mixed-Methods	27	June–August 2020	Limited
Huang et al.^41^	USA (HIC)	Mixed-Methods	1182	January 1, 2020–April 28, 2021	28 (40.6%)
Lis‐kuberka et al.^57^	Poland (HIC)	Quantitative	1196	November 15–December 13, 2021	400 unvaccinated
Middle Income Countries (MIC)					
Ercan et al.^20^	Turkey (MIC)	Quantitative	250	March–April 2021	183 (73.2%)
GoncuAyhan et al.^19^	Turkey (MIC)	Quantitative	300	January 1–February 1, 2021	189 (63%)
Tao et al.^38^	China (MIC)	Quantitative	1392	November 13–27, 2020	315 (22.6%)
Abuhammad et al.^54^	Jordan (MIC)	Quantitative	414	September–October 2021	Not reported
Pairat and Phaloprakarn^26^	Thailand (MIC)	Quantitative	171	July 1–September 30, 2021	67 (39.2%)
Ekmez and Ekmez^56^	Turkey (MIC)	Quantitative	471	October 2020–March 2021	Not reported
Uludağ et al.^39^	Turkey (MIC)	Qualitative	16	October 6–November 13, 2021	12
Qasrawi et al.^21^	Palestine (MIC)	Quantitative	728	October–November 2021	577 (79.3%)
Nguyen et al.^27^	Vietnam (MIC)	Quantitative	651	January–February 2021	258 (39.6%)
Low Income Countries (LIC)					
Mose and Yeshaneh^18^	Ethiopia (LIC)	Quantitative	396	January 1–30, 2021	116 (29.3%)
Nemat et al.^31^	Afghanistan (LIC)	Quantitative	491	July 10–20, 2021	449 (91.4%)
Tefera and Assefaw^15^	Ethiopia (LIC)	Mixed-Methods	702	January 1–30, 2022	77.40%
Aynalem et al.^16^	Ethiopia (LIC)	Mixed-Methods	350	September 1–October 30, 2021	Not reported
Kebede et al.^49^	Ethiopia (LIC)	Quantitative	336	April 7–June 10, 2021	70 (20.1%)
Mixed					
Skjefte et al.^47^	Multiple	Quantitative	17,871	October 28–November 18, 2020	48%
Januszek et al.^32^	Poland (HIC) and Ukraine (MIC)	Quantitative	300	June–August 2021	64.7% in Poland, 83.3% in Ukraine
Naqvi et al.^52^	Multiple	Quantitative	13,105	February–November 2021	8.50%

Summary of extracted articles including country of study, study methods and % of vaccine hesitant pregnant individuals.

### Reasons for vaccine hesitancy

**Fig. 2:**
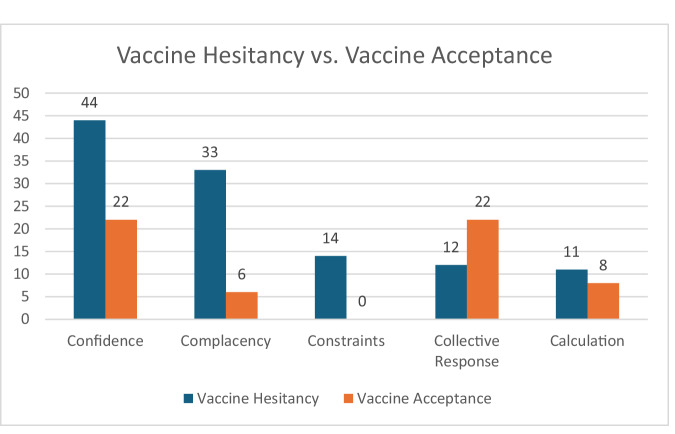
Vaccine hesitancy and vaccine acceptance stratified by 5 C framework. Number of studies in each category of the 5C framework stratified by vaccine hesitancy and vaccine acceptance.

Among extracted articles, reasons given by pregnant persons for COVID-19 vaccine hesitancy were collected and sorted into themes based on the 5Cs framework. All five categories of this framework were represented: Confidence, Complacency, Constraints, Collective Responsibility, and Calculation (Table 2, Fig. 2).

**Table 2:** Vaccine hesitancy vs vaccine acceptance

	Confidence	Complacency	Constraints	Collective responsibility	Calculation
Vaccine hesitancy	Nemat et al.^31^ Rikard-Bell et al.^17^ Ward et al.^34^ Reifferscheid et al.^35^ Tao et al.^38^ Tatarević et al.^37^ Davies et al.^55^ Husain et al.^29^ Mose and Yeshaneh^18^ Tefera and Assefaw^15^ Aynalem et al.^16^ Kebede et al.^49^ Egloff et al.^22^ Schaal et al.^46^ Geoghegan et al.^24^ Colciago et al.^25^ Hosokawa et al.^53^ Abuhammad et al.^54^ Skjefte et al.^47^ Naqvi et al.^52^ Qasrawi et al.^21^ Lis‐kuberka et al.^57^ Nowacka et al.^28^ Januszek et al.^32^ Citu et al.^43^ Citu et al.^50^ Ghamri et al.^42^ Pairat and Phaloprakarn^26^ Ercan et al.^20^ GoncuAyhan et al.^19^ Uludağ et al.^39^ Ekmez and Ekmez^56^ Nguyen et al.^27^ DesJardin et al.^44^ Huang et al.^41^ Levy et al.^14^ Mattocks et al.^36^ Perrotta et al.^48^ Razzaghi et al.^40^ Redmond et al.^45^ Simmons et al.^51^ Sutanto et al.^23^ Sutton et al.^30^ Battarbee et al.^33^	Nemat et al. ^31^ Rikard-Bell et al. ^17^ Ward et al. ^34^ Reifferscheid et al. ^35^ Tao et al. ^38^ Husain et al. ^29^ Mose and Yeshaneh^18^ Tefera and Assefaw^15^ Aynalem et al. ^16^ Kebede et al. ^49^ Egloff et al. ^22^ Colciago et al. ^25^ Hosokawa et al. ^53^ Abuhammad et al.^54^ Skjefte et al. ^47^ Naqvi et al. ^52^ Nowacka et al. ^28^ Januszek et al. ^32^ Citu et al. ^43^ Citu et al. ^50^ Ghamri et al. ^42^ Ercan et al. ^20^ GoncuAyhan et al. ^19^ Nguyen et al. ^27^ Huang et al. ^41^ Levy et al. ^14^ Mattocks et al. ^36^ Perrotta et al. ^48^ Razzaghi et al. ^40^ Redmond et al. ^45^ Simmons et al. ^51^ Sutton et al. ^30^ Battarbee et al. ^33^	Reifferscheid et al. ^35^ Tao et al. ^38^ Tefera and Assefaw^15^ Kebede et al. ^49^ Colciago et al. ^25^ Hosokawa et al. ^53^ Abuhammad et al.^54^ Qasrawi et al. ^21^ Januszek et al. ^32^ GoncuAyhan et al. ^19^ Razzaghi et al. ^40^ Simmons et al. ^51^ Sutanto et al. ^23^ Sutton et al. ^30^	Tefera and Assefaw^15^ Aynalem et al. ^16^ Kebede et al. ^49^ Colciago et al. ^25^ Abuhammad et al.^54^ Naqvi et al. ^52^ Qasrawi et al. ^21^ Citu et al. ^43^ Citu et al. ^50^ GoncuAyhan et al. ^19^ Redmond et al. ^45^ Sutton et al. ^30^	Davies et al. ^55^ Husain et al. ^29^ Tefera and Assefaw^15^ Schaal et al. ^46^ Colciago et al. ^25^ Citu et al. ^50^ Razzaghi et al. ^40^ Redmond et al. ^45^ Simmons et al. ^51^ Sutanto et al. ^23^ Sutton et al. ^30^
Vaccine acceptance	Rikard-Bell et al.^17^ Reifferscheid et al.^35^ Davies et al.^55^ Aynalem et al.^16^ Kebede et al.^49^ Egloff et al.^22^ Geoghegan et al.^24^ Hosokawa et al.^53^ Abuhammad et al.^54^ Qasrawi et al.^21^ Lis‐kuberka et al.^57^ Citu et al.^43^ Citu et al.^50^ Ercan et al.^20^ Uludağ et al.^39^ Ekmez and Ekmez^56^ Huang et al.^41^ Levy et al.^14^ Perrotta et al.^48^ Redmond et al.^45^ Sutton et al.^30^ Battarbee et al.^33^	Kebede et al. ^49^ Schaal et al. ^46^ Hosokawa et al. ^53^ Abuhammad et al.^54^ Huang et al. ^41^ Sutton et al. ^30^		Rikard-Bell et al. ^17^ Reifferscheid et al. ^35^ Davies et al. ^55^ Aynalem et al. ^16^ Kebede et al. ^49^ Egloff et al. ^22^ Schaal et al. ^46^ Geoghegan et al. ^24^ Hosokawa et al. ^53^ Abuhammad et al.^54^ Skjefte et al. ^47^ Qasrawi et al. ^21^ Lis‐kuberka et al. ^57^ Nowacka et al. ^28^ Citu et al. ^50^ Ercan et al. ^20^ Uludağ et al. ^39^ Ekmez and Ekmez^56^ Huang et al. ^41^ Levy et al. ^14^ Perrotta et al. ^48^ Sutton et al. ^30^	Reifferscheid et al. ^35^ Kebede et al. ^49^ Hosokawa et al. ^53^ Lis‐kuberka et al. ^57^ Nowacka et al. ^28^ Citu et al. ^50^ Uludağ et al. ^39^ Sutton et al. ^30^

List of all extracted studies organized into the 5Cs of vaccine behavior framework for both vaccine hesitancy and vaccine acceptance based on data analysis.

#### Confidence

In the context of vaccine hesitancy, a lack of confidence is defined as a lack of trust in vaccines, vaccine delivery systems, and political figures overseeing vaccination programs. A lack of confidence in the vaccination was a major theme of vaccine hesitancy found among all 44 extracted articles^14–57^. Among all 44 studies, concerns regarding the safety of the vaccine, both for themselves and their fetuses, were brought up by pregnant persons. Across 37 articles, pregnant participants feared adverse effects and health complications after getting vaccinated, especially for pregnant persons with chronic medical conditions^14–29,31–38,40,42–45,49,50,52–57^. Specific concerns mentioned by pregnant persons included infertility^15,21,23,28–31^, postpartum hemorrhage^16^, and death^31^. In two studies in the United States, pregnant persons were concerned that harmful ingredients were present in the vaccines^14,23^. Meanwhile, among 33 studies, pregnant participants feared that the vaccine would not be safe for their fetus^14–26,28–39,41,46,47,53–57^. Specific concerns mentioned by pregnant persons included fear of congenital malformations, hearing and visual impairments, preterm birth, and miscarriages^14,16^. Pregnant persons in 5 studies did indicate that they may feel comfortable getting vaccinated post-pregnant or post-breastfeeding^22,29,30,40,41^.

Pregnant persons expressed mistrust in the vaccine due to the lack of data about safety and efficacy, including the lack of representation of pregnant persons in clinical trials, a sentiment seen among 34 studies^15–20,22–31^^,33–35,37–46,48,49,51,53,55^. Among 7 studies, pregnant persons demonstrated discomfort with the rapid development and approval of the COVID-19 vaccine^23,30,35,39,40,47,48^. Pregnant patients specifically in Afghanistan^31^, Romania^43^, and Ethiopia^49^ had concerns about the quality of the vaccine in their respective home countries. Among subjects in 8 studies, there was a fear that the vaccine would cause pregnant persons to get a COVID-19 infection^15,18,19,23,25,30,40,45^. Across 18 studies, mistrust of the medical and political systems that encouraged vaccination was prevalent among pregnant persons^14–16,21,23,28–31,40,41,43–45,47,49–51^. Mistrust of the medical systems can be broken down into lack of belief in the existence of the pandemic and mistrust of medical providers. Disbelief in the pandemic itself was seen among five studies from Romania^50^, Palestine^21^, the United States^41^, Poland^28^, and Ethiopia^15^, meanwhile mistrust of medical providers was a theme only seen in three studies conducted in the United States^23,30,51^. Another theme that was only reiterated among multiple studies conducted in the United States was explicit mistrust of the government, seen among 21–30% of pregnant persons^23,30,40,44,51^. In Romania specifically, 23% of unvaccinated pregnant persons believed that the vaccine was a conspiracy theory^43^. Meanwhile, in Afghanistan^31^ and Ethiopia^16^, some pregnant persons believed that the vaccine had magnetic properties, bad spirits, or mind controlling abilities.

#### Complacency

In the context of vaccine hesitancy, complacency can be defined as believing that the vaccine is not necessary for health or that there are limited perceived risks to the disease for which vaccination is being considered. The theme of complacency was seen across 33 articles^14–20,22,25,27–36,38,40–43,45,47–54^. Some pregnant persons believed that all vaccines were unnecessary. This was most frequently observed in pregnant persons from the United States and Canada across five studies, with up to 13.8% of pregnant persons agreeing with this sentiment^35,40,41,45,48^. A general belief that the COVID-19 vaccine was not necessary was seen mostly among high and upper middle income countries including Australia (6–10%)^17^, the United States (1.4–22%)^14,33^, and Turkey (14.12%)^20^. Among 6 studies, pregnant persons stated they would prefer to utilize other methods besides vaccination to protect themselves from COVID-19^15,16,18,20,30,40^. In a United States study, 21.5% of pregnant persons preferred to use masks and other non pharmaceutical interventions over vaccines^40^. Other methods of protection were also suggested by pregnant persons, including the preparation of spicy foods in Ethiopia^16^. Meanwhile, pregnant persons from Afghanistan^31^, Poland^32^, Ukraine^32^, the United States^30,36,40,41^, England^29^, Romania^50^, Ethiopia^16^, and Vietnam^27^ believed they had already built-up natural immunity to COVID-19 and thus would not require vaccination. There was also the belief, ranging from 2.6% to 13.5% of pregnant participants, that COVID-19 was not a serious disease^19,20,25,40,42,50,51^. In addition to this belief, pregnant persons from a multitude of countries believed that they were not classified as a high-risk group and therefore did not need the vaccination^15,19,22,25,28,34,38,40,42,45,48,51,53,54^.

#### Constraints

In the context of vaccine hesitancy, constraints can be defined as structural barriers–including but not limited to low health literacy, affordability, accessibility, and availability–that prevent vaccination. The theme of Constraints was seen across 14 studies^15,19,21,23,25,30,32,35,38,40,49,51,53,54^. Limited availability of vaccines were barriers for 1.3% of pregnant persons in Poland^32^, 6.7% of pregnant persons in Ukraine^32^, 9.7% of pregnant persons in the United States^40^ and 40.6% of pregnant persons in Palestine^21^. Among two United States studies, 4.1–9.7% of pregnant persons noted they did not know where to get vaccinated^23,40^. In Japan^53^ and Jordan^54^, 16.5–23.5% of pregnant participants stated they did not have enough time to get vaccinated. In China, 3.8% of pregnant persons believed that the vaccination process was unnecessarily complicated^38^. In seven studies, a fear of needles was seen as a barrier to vaccination for 4.2–37.1% of pregnant persons^15,19,21,25,35,40,51^. Costs associated with vaccination were seen as a barrier to vaccination among 2.2–9.6% of pregnant participants in Jordan (2.2%)^54^, Ethiopia (5.06%)^49^, China (7%)^38^, and the United States (9.6%)^40^. In one US based study, there were concerns about the racial inequity in vaccine access, especially among pregnant persons of racial and ethnic minorities^30^.

#### Collective responsibility

In the context of vaccine hesitancy, collective responsibility can be defined as the desire or willingness to protect others. The theme of collective responsibility was seen across 12 studies^15,16,19,21,25,30,43,45,49,50,52,54^. Hesitancy from family members and partners influenced hesitancy among 2.8–47% of pregnant participants^15,19,21,25,30,45^. Meanwhile, hesitancy from friends only influenced hesitancy among a small proportion of pregnant participants in Jordan^54^ and Italy^25^, with less than 10 participants in each study reporting being influenced by friends. Rumors about the COVID-19 that spread through social media also influenced pregnant persons’ hesitancy in two studies, one in the United States^45^ and one in Romania^50^. Religion also played a role in influencing the decision to get vaccinated among pregnant people in South Asia (India, Pakistan, Bangladesh)^52^, Africa (Ethiopia, Kenya, DRC, Zambia)^15,16,49,52^, Latin America (Guatemala)^52^, and Eastern Europe (Romania)^43^.

#### Calculation

In the context of vaccine hesitancy, calculation can be defined as individuals seeking information prior to making a decision about getting a vaccine. The theme of Calculation was present among 11 studies^15,23,25,29,30,40,45,46,50,51,55^. Pregnant participants within these studies noted they did not have sufficient knowledge about the vaccine or COVID-19 to make the decision to get vaccinated^30,40,45,46,50,51^. A lack of recommendation or an explicit recommendation against the COVID-19 vaccine during pregnancy from medical providers influenced 2.1–22.2% of pregnant patients to not get the vaccine^15,25,29,40,45,55^. This phenomenon was mostly seen in England^29,55^, the United States^40,45^, Italy^25^ and Ethiopia^15^ but was more predominant among the three high income countries.

### Reasons for vaccine acceptance

Among extracted articles, reasons given by pregnant persons for COVID-19 vaccine hesitancy were collected and sorted into themes based on the 5Cs framework. Four out of the five categories of this framework were represented: Collective Responsibility, Confidence, Complacency, and Calculation (Table 2, Fig. 2).

#### Collective responsibility

The theme of Collective Responsility was one of the most common themes for vaccine acceptance and was seen across 22 articles^14,16,17,20–22,24,28,30,35,39,41,43,46–49,53–57^. Pregnant persons chose to get the vaccine to protect their fetus, their family, and their community, a theme that was seen across all countries. Protecting their fetuses was most common, and noted across 16 studies^17,20–22,24,30,33,36,39,41,46,49,54–57^. Pregnant persons chose to get vaccinated to protect their fetus from COVID-19 and its consequences, including ICU admissions or death^39^. Pregnant persons also wanted to protect their future pregnancies^30^ and hoped that they would be able to pass immunity to their fetus during pregnancy and breastfeeding^21,30,41,57^. Across 13 studies, pregnant persons chose to get vaccinated because they wanted to protect their family members, especially those who were high-risk and had other comorbidities^17,22,24,30,33,35,39,41,46,49,53–55^. There was also a sense of moral responsibility to protect their community, which was seen across 12 studies^14,17,20,24,30,33,35,43,47–49,53^. Along with this sense of moral responsibility was the desire to return to normal life^21,30,35,43,49^, be able to travel^30,43^, stop wearing a mask^30^, and see family and friends without restrictions^30^. This was seen at a high rate among pregnant persons in Romania (38.5–90.2%)^43^, Canada (57.3%)^35^, and Palestine (36.6%)^21^, while only seen 11% of pregnant persons from Ethiopia^49^.

#### Confidence

The theme of confidence was the other most common theme for vaccine acceptance and was seen across 22 articles^14,16,17,20–22,24,30,33,35,39,41,43,45,48–50,53–57^. Pregnant persons demonstrated a general trust in vaccine safety and effectiveness, as seen across eight studies^14,17,21,24,30,39,49,50^. Five of these studies were conducted in high income countries (Australia^17^, the United States^14,30^, Romania^50^, and Ireland^24^) while only three were conducted in lower- and middle-income countries (Palestine^21^, Turkey^39^, and Ethiopia^49^). Pregnant persons in the United States and Ireland noted that they had increased motivation to accept the vaccine due to the information and data available regarding the safety of COVID-19^14,24,30^. A few of these individuals said that participating in clinical trials or seeing data from pregnant women in clinical trials also increased their likelihood to get the vaccine^14,24,30^. More specifically, pregnant persons believed that the vaccine would protect them from COVID-19 and its complications^14,16,17,20–22,24,30,33,35,39,41,43,48,49,53–57^. Among six studies, many pregnant participants classified themselves as high risk due to chronic illnesses or due to their pregnancy; thus, they wanted to further protect themselves by getting vaccinated^16,20,30,49,53,57^. Another fear among pregnant participants specifically in Ethiopia was the fear of death from COVID-19, which promoted many to get vaccinated^16,49^.

#### Calculation

The theme of calculation was seen across 8 studies^28,30,35,39,43,49,53,57^. The most common source of information and recommendations reported by pregnant persons was healthcare professionals, a trend seen across six studies^28,30,35,43,49,57^. Meanwhile, the influence of family, friends, and social media on vaccine acceptance was only reported by pregnant persons from Japan^53^ and Turkey^39^.

#### Complacency

The theme of complacency was seen across six articles^30,41,46,49,53,54^. Between 10.2% and 88% of pregnant participants in Germany^46^, Japan^53^, Jordan^54^, and the United States^30^ were required to get the vaccine for their jobs, especially if they were healthcare workers. Whereas 6.5% of pregnant participants from an Ethiopian study noted that government recommendations of vaccinations influenced their decision to get vaccinated^49^. Additionally, among two United Sates studies, there was a small number of participants who chose to get vaccinated to avoid re-infection from COVID-19^30,41^ or because they knew someone who had died due to COVID-19 related complications^30^.

## Discussion

Vaccine hesitancy among pregnant persons is complex and multifactorial. A lack of confidence in vaccine safety was the most prevalent theme of hesitancy among pregnant persons. This was largely driven by a lack of access to information about the vaccine as well as mistrust of the vaccine and medical professionals. Meanwhile, vaccine acceptance was mostly driven by pregnant persons’ desires to protect themselves and their loved ones, along with trust in the vaccine and the information provided about the safety of the vaccine.

Both vaccine hesitancy and acceptance were strongly driven by motivations to protect pregnant persons’ families and fetuses. Those who accepted the vaccine believed in the safety of the vaccine and believed that getting vaccinated would protect those around them. Meanwhile, pregnant persons with vaccine hesitancy believed that the vaccine was too experimental, and they were protecting their fetus from potential side effects of the vaccine. Similar trends were seen in a recent study conducted in Japan that found vaccine hesitancy among 51.5% of pregnant women^58^. This, coupled with the fact that most pregnant persons in these studies were open to other vaccinations, shows that COVID-19 vaccination hesitancy is not driven by anti-vaccination sentiments. Rather, it comes from fear of the unknown. In a 2017 systematic review conducted on influenza hesitancy, it was found that hesitancy comes from a lack of knowledge and fear of adverse effects rather than a belief that vaccinations do not work^59^. It is possible that throughout the pandemic, pregnant persons had an increase in confidence in the vaccine as more knowledge was made public regarding the vaccine and its safety profile.

A limited number of studies in this review, concentrated in the United States and Ireland, were the only ones to consider inclusion of pregnant persons in clinical trials as a reason for vaccine acceptance. This suggests that visibility of, and information regarding clinical trials may be more accessible in countries with more resources. However, there is not enough information to support this justification as globally there is a general lack of inclusion of pregnant persons in clinical trials. The lack of pregnant persons in COVID-19 related clinical trials at the start of the pandemic laid the foundation for mixed messaging to pregnant persons. In a systematic review published in the Lancet, 75% of clinical trials for COVID-19 treatments excluded pregnant women^60^. The shift from not recommending the vaccine to explicitly encouraging all doses of the vaccines while pregnant may have increased mistrust and confusion among pregnant persons. However, there are many ethical considerations that must be factored into discussions surrounding inclusion of pregnant persons in clinical trials. A recent publication with a study population of 24 pregnant persons found that 58.3% of them would be unwilling to participate in a hypothetical COVID-19 clinical trial while pregnant^61^. Some of the reasons cited for refusal included concerns that the drug is not safe, fear about the consequences of the drug on themselves or their fetuses, and not enough trust in the current scientific data published^61^. Based on the study, factors that increased willingness to participate in the study include willingness to contribute to science and help other pregnant persons, receiving adequate information from providers regarding necessity and safety of the clinical trial, and support from loved ones^61^.

Efforts have been made to advocate for the inclusion of pregnant persons in clinical trials. As per the 21st Century Cures Act, which became law in December 2016 in the United States, the Task Force on Research Specific to Pregnant Women and Lactating Women was created^62^. Some recommendations from this taskforce included working to increase representation of pregnant persons in clinical trials, working with the FDA to remove pregnant persons from the vulnerable persons categorization, removing regulatory barriers that prevent pregnant persons from being in clinical trials and increasing public awareness surrounding this issue^63^. After the creation of this task force, substantial progress in these efforts have been made. This can be seen via the work of the Pregnancy Research Ethics for Vaccines, Epidemics, and New Technologies (PREVENT) group based out of the Johns Hopkins German Institute of Bioethics^64^. During its first year, the group focused on creating guidelines for inclusion of pregnant persons during the development of the Zika Virus^64^. The most recent example of improvements in this area can be seen by the FDA approval of the RSV vaccine for pregnant persons^65^. This vaccine was approved after two different clinical trials that had a sample population of only pregnant persons^65^.

Vaccine hesitance and acceptance is also contingent upon recommendations from and trust in medical providers. While a lack of recommendation from providers pushed vaccine hesitant pregnant persons away from vaccination, positive recommendations from providers lead to increased rates of vaccine acceptance. The fear of infertility was a common concern across pregnant persons and was a result of the lack of information given as well as the rise in mistrust in scientific information and medical providers. While studies have shown that the COVID-19 vaccine has no impact on fertility^66^, this has not been disseminated properly to the general public. A 2022 systematic review discussed the lack of evidence to prove that the COVID-19 vaccine causes infertility and recommended increased patient education regarding this misinformation^66^. In several of the extracted articles, investigators noted the importance of improved dissemination of information about the benefits and safety of COVID-19 vaccines. Even if information dissemination improves, medical mistrust will also need to be addressed. When evaluating mistrust, mistrust of providers was a theme that was only seen in the United States, while the theme of mistrust in the quality of the vaccine itself was seen among some of the LMICs (e.g., Afghanistan and Ethiopia) represented in our study. The increased prevalence of mistrust of providers among pregnant persons in the United States is likely due to both barriers, including lack of information about the vaccine and lack of access to healthcare, and racial disparities that affect COVID-19 diagnosis, hospitalization, and mortality rates^4^. While this suggests that the causes of mistrust are different among HICs and LMICs, it is important to note that not all studies differentiated between different mechanisms of mistrust. These findings are consistent with the conclusions drawn from literature review conducted in 2021, which also demonstrated that hesitancy and acceptance vary based on geographical location and cultural context^2^.

Approaches to addressing COVID-19 vaccine hesitancy should target improving health literacy among pregnant persons and improving access to timely information about vaccines. Such information should focus on the safety and efficacy of the vaccine for both pregnant persons and their fetuses to assuage common concerns pregnant individuals often have. While the creation of large-scale public health campaigns can improve dissemination of information on a wide scale, it is equally important for resources to be directed towards improving the provider-patient relationship. Improving open-communication skills and focusing on joint-decision making with patients can improve the quality of the provider-patient relationships. There are many common models that are used for patient-centered language, two being the AIDET (Acknowledge, Introduce, Duration, Explanation, Thank you) and RESPECT (Rapport, Empathy, Support, Partnership, Explanation, Cultural Competency, Trust) frameworks^67^. The ACOG discusses these two models and encourages the utilization of patient centered language in clinical encounters^67^. This, in conjunction with consistent COVID-19 vaccine counseling during prenatal care, can increase the likelihood of pregnant patients trusting recommendations made by providers. Above all, the most important recommendation to improve vaccine uptake is to include pregnant persons in clinical trials and allow for autonomous decision making surrounding their participation. Improving representation of pregnant persons in clinical trials will allow for more accurate information on the safety of vaccines and drugs, thus allowing for better communication of health-related information to patients. While broad scaled recommendations can be made to increase COVID-19 vaccine uptake among pregnant persons, it is important to keep in mind that such recommendations will not fit all individuals, especially on a global scale. As the review shows, there were some differences between high income countries and low- and middle-income countries. Therefore, when adapting these recommendations, it is important to consider geographical and cultural contexts and to understand the underlying reasons driving hesitancy among the target population.

Our scoping review had many strengths. This review was performed with a robust and transparent methodology. A broad literature search was conducted via multiple databases with a thorough and comprehensive search stratergy, that had been revised multiple times prior to the final extraction of articles. Our scoping review was conducted via Covidence, which allowed for the use of standardized evaluation tools utilized by our reviewers. Through this review, we were able to evaluate and present a summary of the current literature depicting global vaccine hesitancy trends among pregnant persons.

Due to the nature of a scoping review, it is possible that we were not able to identify and analyze all articles published on the topic of vaccine hesitancy among pregnant persons. As our time frame was only from 2019 to 2022, any studies published after that time frame were not included. We also do not have an equal number of studies from HIC and LIMC studies, and a large majority of our cases come from the USA. Thus, these findings cannot be broadly generalized.

Overall, levels of COVID-19 vaccine hesitancy among pregnant persons is high. Vaccine hesitancy is primarily driven by fear of unknown side effects of the vaccine upon pregnant persons and their fetuses, along with a lack of information and medical mistrust. Some differences can be seen between HIC and LMIC regarding the motivations that guide vaccine hesitancy, showing that a single solution cannot be applied to all who are vaccine hesitant. General strategies that can be utilized to improve vaccine uptake, including advocating for the inclusion of pregnant persons in clinical trials and incorporating consistent COVID-19 vaccine counseling based on information from the appropriate governing bodies and professional societies during prenatal appointments are feasible solutions to this growing problem.

## Methods

A scoping review methodology was chosen to interrogate existing literature on COVID-19 vaccine hesitancy or attitudes towards the COVID-19 vaccine among pregnant persons and to uncover the remaining gaps to inform future research efforts. Findings of this scoping review are reported based on the Preferred Reporting Items for Systematic Reviews and Meta-Analyses (PRIMSA) guidelines. A literature search was conducted across PubMed, Embase, CINHAL, and SCOPUS on October 2nd, 2022. Inclusion criteria included articles published in English between 2019 and 2022 focused on reasons for COVID-19 vaccine hesitancy or attitudes toward COVID-19 vaccination among pregnant people. Articles were included if they focused on either the primary vaccine series or subsequent booster vaccines. Articles not focused on pregnant persons and COVID-19 vaccine hesitancy were excluded, along with commentaries, review articles, meta-analyses, and non-peer reviewed studies. The search strategy focused on four main concepts: Pregnancy, COVID-19, Vaccine, and Hesitancy. Boolean operators (AND, OR) were used along with database specific subject headings (e.g. MeSh terms in PubMed).

Three independent reviewers performed initial citation screenings with interrater reliabilities of 0.95 (*k* = 0.78), 0.91 (*k* = 0.64), 0.97 (*k* = 0.87). Two independent reviewers reviewed citations during the full-text extraction stage with an interrater reliability of 0.8 (*k* = 0.57). The following data were extracted from each study: study information (location, methodology, sample size, study timeline); demographic information (age, race/ethnicity, gravidity, and parity status); quantification of vaccine hesitant and non-hesitant participants; vaccination status; definition of vaccine hesitance and vaccine acceptance; reasons for vaccine hesitance and vaccine acceptance; correlates and predictors of vaccine hesitance and acceptance; recommendations to improve COVID-19 vaccine uptake among pregnant persons; acceptance and hesitance of other vaccines.

**Fig. 3:**
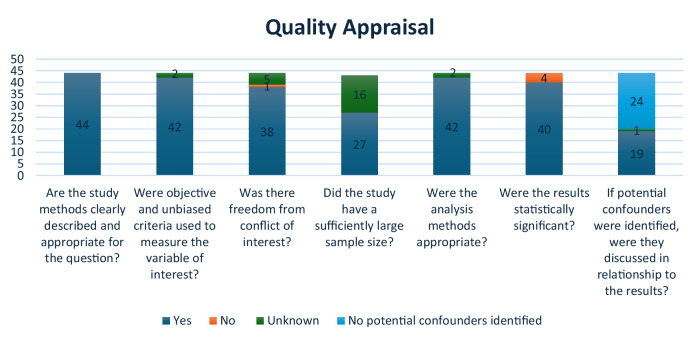
Quality appraisal. Results of completed quality appraisals for all 44 extracted articles.

Quality appraisal, conducted via Covidence, looked at appropriateness of the study methodology, objectiveness of data, conflict of interest, sample size, analysis methodology, statistically significant results, and identification of potential confounders. Results of the quality appraisal can be found in Fig. 3.

Data analysis was guided by the 5 C model, a behavioral model proposed by Corenlia Betsch and her team which has been commonly used to assess vaccine acceptance and hesitancy^68^. The 5 Cs in this model are Confidence, Complacency, Constraints, Calculation and Collective Responsibility, which are defined below^68^. Through this model, the psychological underpinnings of vaccine hesitancy and acceptance can be better understood. This model was chosen for data analysis as it is a commonly recognized methodology that has been used to assess vaccine acceptance and hesitancy with other vaccines as well. This model was used to categorize and report the responses from pregnant participants across included articles.

**Confidence:** Trust in vaccine safety and efficacy; Trust in vaccine delivery systems; Trust in policy makers.

**Complacency:** Perceived risks of vaccine preventable diseases; Vaccination not deemed necessary or important.

**Constraints:** Accessibility, availability, and affordability of vaccines; Health literacy; Other structural barriers that prevent vaccine uptake.

**Calculation:** Individuals seeking information about vaccination prior to decision making.

**Collective responsibility:** The willingness to protect others and influence from others.

## Data Availability

All relevant data are available from the authors.
